# Definition, Incidence, and Challenges for Assessment of Hyperprogressive Disease During Cancer Treatment With Immune Checkpoint Inhibitors: A Systematic Review and Meta-analysis

**DOI:** 10.1001/jamanetworkopen.2021.1136

**Published:** 2021-03-24

**Authors:** Hyo Jung Park, Kyung Won Kim, Sang Eun Won, Shinkyo Yoon, Young Kwang Chae, Sree Harsha Tirumani, Nikhil H. Ramaiya

**Affiliations:** 1 Asan Image Metrics, Asan Medical Center, Department of Radiology and Research Institute of Radiology, University of Ulsan College of Medicine, Seoul, Republic of Korea; 2 Asan Medical Center, Department of Oncology, University of Ulsan College of Medicine, Seoul, Republic of Korea; 3 Feinberg School of Medicine, Robert H. Lurie Comprehensive Cancer Center, Department of Medicine, Northwestern University, Chicago, Illinois; 4 University Hospitals Cleveland Medical Center, Department of Radiology, Case Western Reserve University, Cleveland, Ohio

## Abstract

**Importance:**

Hyperprogressive disease (HPD) is a recognized pattern of rapid tumor progression during immune checkpoint inhibitor (ICI) treatment. Definitions of HPD have not been standardized, posing the risk of capturing different tumoral behaviors.

**Objectives:**

To provide a systematic summary of definitions and the incidence of HPD in patients undergoing ICI treatment and discuss the challenges of current assessment of HPD.

**Data Sources:**

Articles that evaluated HPD published before March 3, 2020, were identified from MEDLINE and EMBASE.

**Study Selection:**

Clinical trials and observational studies providing the incidence and definition of HPD from patients with cancer treated with ICIs.

**Data Extraction and Synthesis:**

Factors included in the analysis comprised authors, year of publication, cancer type, ICI type, number of previous treatment lines, definition of HPD, time frame used to assess HPD, number of patients with HPD, onset of HPD, and prognosis of patients with HPD. Quantitative and qualitative syntheses for the incidence of HPD were performed.

**Main Outcomes and Measures:**

Definitions of HPD were categorized and the range of incidence of HPD was evaluated. Subgroup analysis on the incidence of HPD according to the category was performed and the challenges associated with current HPD assessment were evaluated.

**Results:**

Twenty-four studies with 3109 patients were analyzed. The incidence of HPD varied from 5.9% to 43.1%. The definitions were divided into 4 categories based on the calculation of tumor growth acceleration: tumor growth rate ratio (pooled incidence of HPD, 9.4%; 95% CI, 6.9%-12.0%), tumor growth kinetics ratio (pooled incidence, 15.8%; 95% CI, 8.0%-23.7%), early tumor burden increase (pooled incidence, 20.6%; 95% CI, 9.3%-31.8%), and combinations of the above (pooled incidence, 12.4%; 95% CI, 7.3%-17.5%). Hyperprogressive disease could be overestimated or underestimated if the assessment was limited to tumor growth rate or tumor growth kinetics ratio, target lesions, or response evaluation criteria in solid tumors (RECIST)–defined progressors, or if the assessment time frame conformed to RECIST. Study results on clinical outcome were heterogeneous on discriminating patients with HPD from those with natural progressive disease.

**Conclusions and Relevance:**

Definitions of HPD appear to be diverse, with the incidence of HPD varying from 5.9% to 43.1% across studies examined in this meta-analysis. Varying incidence and definitions of HPD indicate the need for establishing its uniform and clinically relevant criteria based on currently available evidence.

## Key Points

**Question:**

What are the definition, incidence, and challenges associated with the current assessment of hyperprogressive disease among patients receiving immune checkpoint inhibitor therapy for cancer?

**Findings:**

In this systematic review and meta-analysis of 24 studies including 3109 patients, the definition of hyperprogressive disease varied across studies and was divided into 4 categories: tumor growth rate ratio, tumor growth kinetics ratio, early tumor burden increase, and combinations of these categories. The incidence of hyperprogressive disease varied from 6% to 43%.

**Meaning:**

Varying definitions and incidences of hyperprogressive disease indicate the need for establishing uniform and clinically relevant criteria based on currently available evidence.

## Teaser

The systematic review and meta-analysis examines differences in the criteria and definitions used for hyperprogressive disease in studies assessing patients receiving immune checkpoint inhibitors for cancer.

## Introduction

In the era of cancer immunotherapy, immune checkpoint inhibitors (ICIs) targeting cytotoxic T-lymphocyte–associated antigen 4 (CTLA-4) and programmed cell death protein 1 (PD-1) or its ligand (PD-L1) are used across various cancer types in clinical trials and in practice.^1^ However, atypical patterns of response, such as pseudoprogression and hyperprogressive disease (HPD), have been observed in cancers treated with ICIs.^2,3^

In general, HPD refers to the unexpected rapid acceleration of tumor growth occurring in a subset of patients treated with ICIs.^1,2^ In contrast to the pseudoprogression in which tumor burden increase is provoked by an inflammatory reaction and followed by tumor response,^3^ HPD is thought to be caused by tumor growth prompted by an idiosyncratic effect of ICIs as enhancers of tumor progression.^4^

Previous studies have reported that patients with HPD showed shorter overall survival (OS) or progression-free survival compared with patients with natural progressive disease (PD).^5,6,7^ Thus, discrimination of the particularly deleterious HPD from the natural PD might be important but is particularly challenging in daily clinical practice. To our knowledge, there has been no unified definition of HPD or summarized data on its incidence. Heterogeneous assessment of HPD poses the risk of capturing different tumoral behaviors.^4^

So far, there have been scattered individual studies exploring HPD with varying definitions.^5,6,7,8,9,10,11,12,13,14,15,16,17,18,19,20,21,22,23,24,25,26,27,28^ To our knowledge, no attempt has yet been made to generate a more evidence-based systematic summary about definitions and incidence of HPD. Therefore, we aimed to perform a systematic review to summarize the proposed definitions of HPD and reported incidence of HPD, which may help provide a more standardized diagnosis of HPD in patients receiving ICI treatment.

## Methods

A comprehensive search of MEDLINE and EMBASE was conducted to identify relevant studies published before March 3, 2020. The following search terms were used: *immunotherapy*, *checkpoint*, *check-point*, *check*, *PD1*, *PD-L1*, or *CTLA-4*, *ipilimumab*, *nivolumab*, *pembrolizumab*, *atezolizumab*, *avelumab*, or *durvalumab*, and *hyperprogression* or *hyperprogressive*. There was no limit to the start date or type of language. A detailed search strategy is provided in eTable 1 in the Supplement. The bibliographies of articles were screened for potentially suitable articles. This study followed the Preferred Reporting Items for Systematic Reviews and Meta-analyses (PRISMA) reporting guideline^29^ and Meta-analysis of Observational Studies in Epidemiology (MOOSE) reporting guideline^30^ for study selection, data collection and synthesis, assessment of bias, and sensitivity analysis.

Based on the PICOS (population, intervention, comparison, outcome, study design) approach,^31^ we selected studies fulfilling the following criteria: (1) population as patients with solid malignant tumors, (2) intervention as ICI treatment, (3) outcome as incidence and definition of HPD, and (4) study design as clinical trials and observational studies, either prospective or retrospective. The exclusion criteria were (1) other publication types (ie, conference abstracts, case reports, letters, or reviews), (2) studies with fewer than 9 patients, and (3) studies that included patients with primary brain tumors or hematologic malignancies. After the database searches, an initial screening of all titles and abstracts was conducted. Subsequently, all potentially relevant studies were evaluated based on full-text reviews. Studies were excluded if they failed to meet the inclusion criteria described above. Two of us (H.J.P. and K.W.K.) independently selected literature eligible for review. Disagreements between the 2 reviewers occurred regarding 2 studies^27,28^ and were resolved by consensus with 1 of us (S.Y.).

Although conference abstracts were excluded from the main systematic review and meta-analysis, we selected abstracts that contained sufficient information. The detailed information of selected abstracts is provided in eFigure 1 in the Supplement.

The following data were extracted into standardized forms: (1) study characteristics (authors, year of publication, study design, and sample size), (2) demographic and clinical characteristics (cancer type, ICI type, number of previous treatment lines, the time between prebaseline computed tomographic [CT] scan and baseline CT scan before treatment onset, and the time between baseline CT scan and first follow-up CT scan for response evaluation), and (3) outcome characteristics (definition of HPD, number of patients with HPD, onset of HPD, and prognosis of patients with HPD).

Data extraction was performed by 2 of us (H.J.P. and K.W.K.) independently. To categorize the definitions of HPD, after listing all HPD definitions suggested to date, we identified similarities and differences among the definitions and placed them in 4 categories.

Two of us (H.J.P. and K.W.K.) also independently reviewed the study quality and risk of bias in individual studies using the Newcastle-Ottawa Scale (NOS), which allows a total score of 9 points or fewer (9 indicates the highest quality) regarding the aspects of selection (maximum, 4 points), comparability (maximum, 2 points), and outcomes (maximum, 3 points) of study cohorts.^32^ Any discrepancy was resolved by discussion with 1 of us (S.Y.).

To explore the applicability, appropriateness, and clinical relevance of HPD definitions proposed in the included studies, we addressed the following questions:

1. Can HPD definitions be applied to most patients during ICI treatment?
2. Is there any risk of overestimation or underestimation of HPD based on tumor kinetics assessment?
3. Can HPD definitions appropriately reflect the change in overall tumor burden?
4. Is PD defined by tumor response evaluation criteria necessary to define HPD?
5. Is a time frame (ie, between prebaseline and baseline CT scan before treatment onset and time between baseline and first follow-up CT scan) needed to define HPD, and if so, what is the optimal time frame?
6. Is HPD associated with clinical outcome by discriminating patients with HPD from those with natural PD?

### Statistical Analysis

The pooled incidence of HPD was obtained by a random-effects model with an inverse-variance weighting model.^33^ Heterogeneity was evaluated using the Higgins inconsistency index (*I*^2^) test and Cochran *Q* test, and *I*^2^ greater than 50% or *P* < .10 values from a *Q* test indicated significant heterogeneity.^34,35,36,37^ Publication bias was assessed by a funnel plot and the Begg test.^38^ To test the robustness of the meta-analysis results, sensitivity analyses were performed to recalculate the pooled incidence of selected studies based on an NOS score greater than or equal to 7 and recalculate the pooled incidence after excluding each study (ie, leave-1-out method). Subgroup analyses were performed to calculate the pooled HPD incidence according to each category of the HPD definition with *P* value correction using the Tukey method to account for multiple comparisons; with 2-sided, unpaired testing, *P* < .05 was considered significant. Statistical analyses were performed using the metafor package in R, version 4.0.2 (R Foundation for Statistical Computing).^33^

## Results

**Figure 1.**
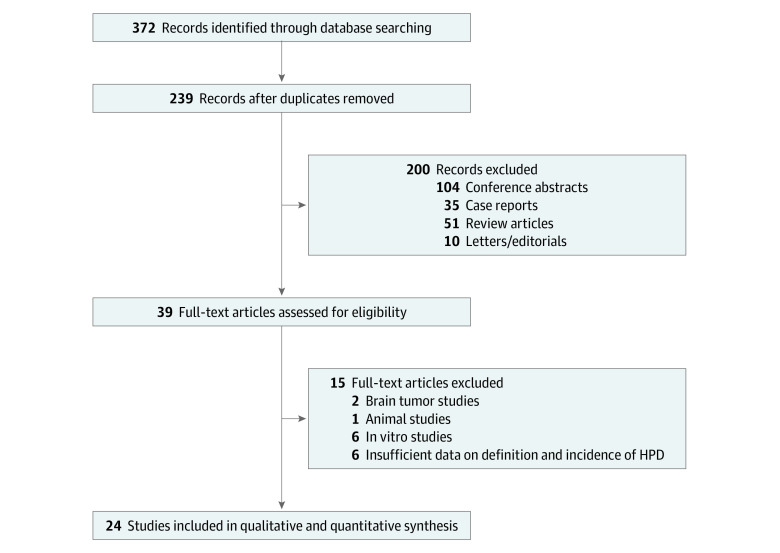
Study Selection Process HPD indicates hyperprogressive disease.

A total of 3109 patients were included (Figure 1). The characteristics of the 24 included studies^5,6,7,8,9,10,11,12,13,14,15,16,17,18,19,20,21,22,23,24,25,26,27,28^ are summarized in Table 1. There were 17 retrospective studies, 5 retrospective studies of clinical trial data, and 2 prospective studies. Nine studies included various tumor types (≥3 tumor types in each study). In 15 tumor-specific studies, the most common tumor was non–small-cell lung cancer (8 studies). The number of previous treatment lines was heterogeneous, ranging from 0 to 9.

**Table 1.** Characteristics of the Studies Included in the Meta-analysis

Source	Study design	Tumor	Treatment	No. of previous treatment lines	HPD definition	No. of patients	Incidence of HPD, No./No. (%)	Treatment period		Prognostic outcome of HPD	
								Pre	Post	HPD vs non-HPD	HPD vs PD without HPD
Champiat et al,^5^ 2017	Retrospective analysis of clinical trial data	Melanoma, lung cancer, RCC, colorectal cancer, urothelial cancer, and others	PD-1 or PD-L1 inhibitor monotherapy	0-9	PD by RECIST 1.1 at first evaluation and post-TGR/pre-TGR≥2	131	12/131 (9.2)	2 wk to 3 mo	6-8 wk	OS: HR, 2.6 (*P* < .001)	OS: 4.6 vs 7.6 mo (*P* = .19)
Kato et al,^8^ 2017	Retrospective	NSCLC, head and neck cancer, cutaneous SCC, melanoma, RCC	PD-1 or PD-L1 inhibitor monotherapy	NA	TTF<2 mo, >50% increase in tumor burden vs preimmunotherapy imaging, and post-TGK/pre-TGK>2	102	6/102 (5.9)	<2 mo	NA	NA	NA
Saâda-Bouzid et al,^9^ 2017	Retrospective	SCC of head and neck	PD-1 and/or PD-L1 inhibitors	0-≥2	Post-TGK/pre-TGK≥2	34	10/34 (29.4)	NA	NA	OS: 6.1 vs 8.1 mo (*P* = .77); PFS: 2.5 vs 3.4 mo (*P* = .003)	NA
Ferrara et al,^6^ 2018	Retrospective	NSCLC	PD-1 and/or PD-L1 inhibitors	0-8	PD by RECIST 1.1 at first evaluation and TGR change >50% per month	406	56/406 (13.8)	2-6 wk	2-6 wk	NA	OS: HR, 2.18 (*P* < .001)
Abbas et al,^28^ 2019	Retrospective	Urothelial cell carcinoma (n = 3) and melanoma (n = 1)^a^	PD-1 or PD-L1 inhibitor monotherapy	NA	>2-fold increase in tumor size	50	4/50 (8.0)	NA	>2 mo	NA	NA
Aoki et al,^10^ 2019	Retrospective	AGC	Nivolumab with or without irinotecan	≥2	Post-TGR/pre-TGR≥2	34	10/34 (29.4)	NA	NA	OS: HR, 4.7 (*P* = .002); PFS: HR, 3.4 (*P* = .004)	OS: HR, 2.1 (*P* = .17); PFS: HR, 1.1 (*P* = .76)
Hwang et al,^11^ 2020	Retrospective	Urothelial carcinoma, RCC	PD-1 or PD-L1 inhibitor monotherapy (70.9%) or with targeted agents (29.1%)	0-1	(1) TTF<2 mo, >50% increase in the tumor burden, and post-TGR/pre-TGR>2 or (2) ≥10 new measurable lesions	203	13/203 (6.4)	4-8 wk	6-8 wk	OS and PFS significantly shorter in patients with vs without HPD	OS: 3.5 vs 7.3 mo (*P* < .001)
Ji et al,^12^ 2019	Retrospective analysis of clinical trial data	Gastric cancer, esophageal cancer, colorectal cancer, liver cancer, pancreatic cancer, ampulla cancer	PD-1 or PD-L1 inhibitor monotherapy or combined with CTLA-4 inhibitor	≥1	Post-TGK/pre-TGK≥2	25	5/25 (20.0)	NA	NA	NA	NA
Kamada et al,^13^ 2019	Retrospective	AGC	Nivolumab	NA	TTF<2 mo, >50% increase in tumor burden vs pretreatment imaging, and post-TGK/pre-TGK>2	36	4/36 (11.1)	NA	<2 mo	NA	NA
Kanjanapan et al,^14^ 2019	Retrospective analysis of clinical trial data	Head and neck cancer, gynecological cancer, lung cancer, gastrointestinal cancer, genitourinary cancer, and others	PD-1 and/or PD-L1 inhibitors (89%), other checkpoint inhibitors (3%) or costimulatory molecules (8%)	<4 (87%), ≥4 (13%)	PD by RECIST 1.1 at first evaluation and post-TGR/pre-TGR>2	182	12/182 (6.6)	2 wk to 3 mo	NA	OS: HR, 1.7 (*P* = .11); PFS: 3.7 (*P* < .001)	NA
Kim et al,^7^ 2019	Retrospective	NSCLC	PD-1 or PD-L1 inhibitor monotherapy	0-8	Post-TGR/pre-TGR>2 or post-TGK/pre-TGK>2 in patients with PD by RECIST 1.1 at first evaluation	237	45/237 (19.0)	12 wk	12 wk	OS and PFS significantly shorter in patients with HPD	OS: 1.6 vs 6.7 mo (*P* < .001); PFS: 0.6 vs 1.6 mo (*P* < .001)
Kim et al,^15^ 2019	Retrospective	NSCLC	PD-1 or PD-L1 inhibitor monotherapy	1-7	Definition 1: TTF<2 mo, post-TGR/pre-TGR>2, and volume increase of 50% vs baseline^b^;	335	48/335 (14.3)	2-3 mo	Approximately 2 mo	OS: 4.7 vs 7.9 mo (*P* = .009)	NA
					Definition 2: post-TGR/pre-TGR>2	335	44/335 (13.1)	2-3 mo	Approximately 2 mo	OS: 5.2 vs 7.1 mo (*P* = .23)	NA
Lo Russo et al,^16^ 2019	Retrospective	NSCLC	PD-1 and/or PD-L1 inhibitors	0-2	Fulfilling ≥3 of the following: (1) TTF<2 mo, (2) ≥50% increase of tumor burden between baseline and first evaluation, (3) ≥2 new lesions in an organ already involved between baseline and first evaluation, (4) disease spread to a new organ between baseline and first evaluation, and (5) decrease in ECOG performance status ≥2 during the first 2 mo of treatment	152	39/152 (25.7)	NA	8 wk	OS: 4.4 (95% CI, 3.4-5.4) vs 17.7 mo (95% CI, 13.4-24.1)	OS: 4.4 (95% CI, 3.4-5.4) vs 8.7 mo (95% CI, 5.3-13.4)
Lu et al,^17^ 2019	Prospective	Gastric cancer, esophageal cancer, colorectal cancer, and others	PD-1 or PD-L1 inhibitor monotherapy or combined with CTLA-4 inhibitor	≥1	Post-TGK/pre-TGK≥2	56	5/56 (8.9)	NA	NA	OS: 3.6 vs 11.4 mo (*P* < .01); PFS: 1.4 vs 4.2 mo (*P* < .001)	NA
Matos et al,^18^ 2020	Retrospective analysis of clinical trial data	Melanoma, NSCLC, colorectal cancer, gastric cancer, breast cancer, head and neck cancer, cervical cancer, bladder cancer, and others	PD-1 or PD-L1 inhibitor monotherapy or combined with CTLA-4 inhibitor	NA	Definition 1: PD by RECIST at first 8 wk after treatment initiation and minimum increase in the measurable lesions of 10 mm plus: (1) 40% increase in STL vs baseline and/or (2) 20% increase in STL vs baseline plus the appearance of new lesions in at least 2 different organs	270	29/270 (10.7)	3 mo to 2 wk	8 wk	NA	OS: 5.23 vs 7.33 mo (HR, 1.73; *P* = .04)
					Definition 2: post-TGR/pre-TGR>2	221	14/221 (6.3)	3 mo to 2 wk	8 wk	NA	OS: 4.2 vs 6.27 mo (HR, 1.4 *P* = .35)
Sasaki et al,^19^ 2019	Retrospective	AGC	Nivolumab	≥1	Post-TGK/pre-TGK>2 and >50% increase in tumor burden vs that at pretreatment imaging	62	13/62 (21.0)	NA	<3 mo	OS: HR, 9.16 (*P* < .001); PFS: HR, 4.82 (*P* < .001)	NA
Scheiner et al,^20^ 2019	Retrospective	HCC	PD-1 inhibitor monotherapy	≥1	PD by RECIST at first evaluation with a TGR change >50% per month	52	4/52 (7.7)	NA	6-12 wk	NA	NA
Ten Berge et al,^21^ 2019	Retrospective	NSCLC	Nivolumab	≥1	Post-TGR/pre-TGR>2	58	4/58 (6.9)	Median, 1.8 mo	6-8 wk	OS: 2.3 vs 12.3 mo (*P* = .04)	NA
Tunali et al,^22^ 2019	Retrospective analysis of clinical trial data	NSCLC	PD-1 or PD-L1 inhibitor monotherapy or combined with CTLA-4 inhibitor	NA	Post-TGR/pre-TGR>2, PD by RECIST at first evaluation, and TTF<2 mo	228	15/228 (6.6)	2 wk to 3 mo	4 wk to 2 mo	OS significantly shorter in patients with HPD	NA
Arasanz et al,^23^ 2020	Prospective	NSCLC	PD-1 and/or PD-L1 inhibitors	≥1	PD by irRC at first evaluation with a ≥2-fold increase of TGR after immunotherapy	56	10/56 (17.9)	NA	NA	OS: 3.2 vs 12.6 mo (*P* = .006)	PFS significantly shorter in patients with HPD (*P* = .04)
										PFS: 1.4 vs 2.5 mo (*P* < .001)	
Forschner et al,^24^ 2020	Retrospective	Acral and mucosal melanoma	PD-1 and/or PD-L1 inhibitors	NA	PD by RECIST and tumor burden increased by >50%	51	22/51 (43.1)	NA	Median, 11 wk	Disease-specific survival significantly shorter in patients with HPD	NA
Petrioli et al,^25^ 2020	Retrospective	Lung cancer, head and neck cancer, kidney cancer, bladder cancer, and HCC	Nivolumab	1-2	Post-TGR/pre-TGR≥2	47	3/47 (6.4)	3 wk	<3 wk	NA	NA
Refae et al,^26^ 2020	Retrospective	NSCLC, head and neck SCC, melanoma, RCC, and others	PD-1 or PD-L1 inhibitor monotherapy	0-≥4	Post-TGR/pre-TGR>2	80	11/80 (13.8)	NA	NA	OS: HR, 6.1 (*P* = .003)	NA
Ruiz-Patiño et al,^27^ 2020	Retrospective	NSCLC	PD-1 or PD-L1 inhibitor monotherapy or combined with CTLA-4 inhibitor or chemotherapy	0-≥3	PD by RECIST 8 wk after initiation of treatment	222	44/222 (19.8)	NA	NA	NA	NA

Abbreviations: AGC, advanced gastric cancer; CTLA, cytotoxic T-lymphocyte–associated antigen 4; ECOG, Eastern Cooperative Oncology Group; HCC, hepatocellular carcinoma; HPD, hyperprogressive disease; HR, hazard ratio; irRC, immune-related response criteria; NA, not available; NSCLC, non–small cell lung cancer; OS, overall survival; PD, progressive disease; PD-1, programmed cell death protein 1; PD-L1, PD-1 ligand; PFS, progression-free survival; post, posttreatment assessment period; pre, pretreatment assessment period; RCC, renal cell carcinoma; RECIST, response evaluation criteria in solid tumors; SCC, squamous cell carcinoma; STL, sum of target lesions; TGK, tumor growth kinetics; TGR, tumor growth rate; TTF, time to failure.

^a^ Cancer types of other patients were not reported.

^b^ Tumor growth rate was obtained by the computed tomographic volumetry assessment, and the number of lesions to be analyzed was not limited (any detected lesion was included in the analysis).

Thirteen studies compared OS between patients with HPD and those without HPD, and in 12 studies, patients with HPD showed significantly shorter OS. In 6 studies that reported the comparison between patients with HPD and those with natural PD, patients with HPD showed significantly shorter OS than those without. In terms of progression-free survival, all studies with available data reported significantly shorter progression-free survival in patients with HPD than in patients without HPD as well as those with natural PD.

Among the 104 conference abstracts that were excluded from the main systematic review and meta-analysis, 29 abstracts had relevant information on HPD in patients treated with ICIs. Detailed information is available in eTable 2 in the Supplement.

The NOS scores allocated for each study ranged from 4 to 9 points, with a mean value of 7 points (eTable 3 in the Supplement). Among 24 studies, 22 were awarded 3 points and 2 were awarded 4 points in the selection of cohorts. In the comparability of cohorts, 18 studies were awarded 1 or 2 points, and 6 studies did not get any points. In the outcomes, 18 studies were awarded 3 points and 6 studies were given 1 point. Overall, there were 15 studies with NOS scores greater than or equal to 7.

The definitions of HPD substantially varied and were categorized according to the calculation of tumor growth acceleration as follows: category 1, tumor growth rate (TGR) ratio to compare the speed of increase in tumor volume before and after treatment; category 2, tumor growth kinetics (TGK) ratio to compare the speed of increase in tumor size before and after treatment; category 3, early tumor burden increase between baseline imaging and the first time point after treatment; and category 4, combinations of these categories (Table 2). The categories could be further divided according to the consideration of new lesions and time to failure. Tumor growth rate was defined as the percentage of increase in tumor volume per month and was calculated as follows: TGR = 100 [exp(TG) – 1], where TG is 3-log (St/S0) and St and S0 are the tumor sizes at times t and 0, respectively, defined as the sum of the longest diameters of the target lesions as per response evaluation criteria in solid tumors (RECIST) 1.1.^39,40^ Tumor growth kinetics was defined as the change in the tumor size per unit of time (millimeters per day) and was calculated as follows: TGK = (St – S0)/(t – t0).^41^

**Table 2.** Categorization of HPD Definitions Proposed in the Included Studies According to the Concept of Tumor Growth Acceleration

Category	Parameters of tumor growth acceleration	Evaluating lesions	Consideration of TTF	Source
1	TGR ratio (post-TGR/pre-TGR)^a^	Target lesions only	TTF <2 mo	Tunali et al,^22^ 2019
			None	Champiat et al,^5^ 2017; Ferrara et al,^6^ 2018; Aoki et al,^10^ 2019; Kanjanapan et al,^14^ 2019; Scheiner et al,^20^2019; Ten Berge et al,^21^ 2019; Matos et al,^18^ 2020^c^; Kim et al,^15^ 2019^c^; Arasanz et al,^23^ 2020; Petrioli et al,^25^ 2020
2	TGK ratio (post-TGK/pre-TGK)^b^	Target lesions only	None	Saâda-Bouzid et al,^9^ 2017; Ji et al,^12^ 2019; Lu et al,^17^ 2019; Refae et al,^26^ 2020
3	Early tumor burden increase	Target lesions only	TTF <2 mo	Ruiz-Patiño et al,^27^ 2020
			None	Abbas et al,^28^ 2019; Forschner et al,^24^ 2020
		Target and new lesions	TTF <2 mo	Lo Russo et al,^16^ 2019; Matos et al,^18^ 2020^c^
4	Both TGK ratio and early tumor burden increase	Target lesion only	TTF <2 mo	Kato et al,^8^ 2017; Kamada et al,^13^ 2019
			None	Sasaki et al,^19^ 2019
	Both TGR ratio and early tumor burden increase	Target and new lesions	TTF <2 mo	Hwang et al,^11^ 2020; Kim et al,^15^ 2019^c^
	Either TGR ratio or TGK ratio	Target lesion only	None	Kim et al,^7^ 2019

Abbreviations: HPD, hyperprogressive disease; TGK, tumor growth kinetics; TGR, tumor growth rate; TTF, time to failure.

^a^ Post-TGR/pre-TGR calculated by tumor volume.

^b^ Post-TGK/pre-TGK calculated by tumor size.

^c^ Matos et al^18^ and Kim et al^15^ compared 2 HPD definitions.

In 20 of 24 studies (83.3%), calculation of tumor growth acceleration (TGR ratio and/or TGK ratio) was required to define HPD. Only 4 studies considered new lesions in addition to target lesions for defining HPD. In 12 studies, the time between baseline and first follow-up CT scan was within 2 months (time to failure of <2 months was required in 8 studies and not in 4 studies), in 6 studies the time between baseline and first follow-up CT scan was within 3 months according to RECIST, and in 6 studies the time between baseline and first follow-up CT scan was not defined.

**Figure 2.**
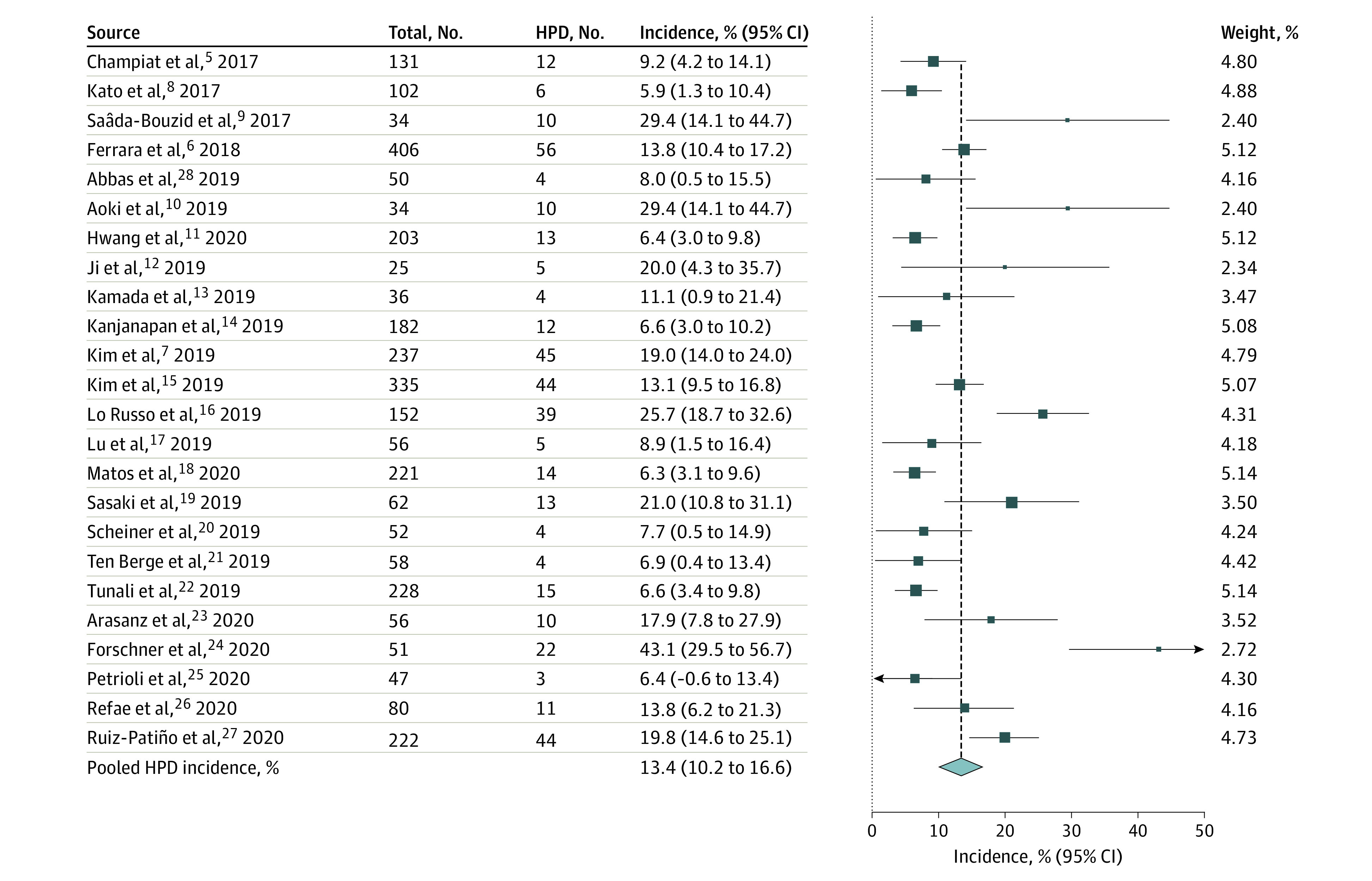
Overall Pooled Incidence of Hyperprogressive Disease (HPD) The pooled incidence of HPD was 13.4% (95% CI, 10.2%-16.6%). Significant heterogeneity was observed (*I*^2^ = 87.6%; *P* < .001).

The incidence of HPD varied from 5.9% to 43.1%. The pooled incidence of HPD was 13.4% (95% CI, 10.2%-16.6%) (Figure 2). Significant heterogeneity was observed (*I*^2^ = 87.6%; *P* < .001). If studies provided 2 incidences of HPD from different definitions, we used the TGR-based definition to obtain the pooled result (Kim et al^15^ and Matos et al^18^). Visual inspection of the funnel plot (eFigure 2 in the Supplement) revealed asymmetry, and a significant publication bias was noted according to the Begg test (*P* = .003).

As a sensitivity analysis, we calculated the pooled incidence of HPD in studies with an NOS score of 7 or higher (n = 15), which was 15.9% (95% CI, 11.3%-20.6%) (eFigure 3 in the Supplement). The sensitivity analysis using a leave-1-out method demonstrated that the pooled incidence of HPD ranged from 12.2% to 13.8%. These sensitivity analyses demonstrated the robustness of the pooled incidence of HPD.

### Subgroup Analysis

The pooled incidences of HPD in the subgroups classified according to the definition of HPD and the types of tumor are provided in eTable 4 in the Supplement. Regarding the subgroup analysis according to the definition of HPD, the pooled HPD incidence of category 3 was the highest (early tumor burden increase, 20.6%; 95% CI, 9.3%-31.8%), followed by category 2 (TGK ratio, 15.8%; 95% CI, 8.0%-23.7%), category 4 (combination, 12.4%; 95% CI, 7.3%-17.5%), and category 1 (TGR ratio, 9.4%; 95% CI, 6.9%-12.0%) without significant differences between subgroups (*P* ≥ .06).

As to the subgroup analysis according to the tumor types, the pooled incidence of HPD was 15.0% (95% CI, 10.5%-19.5%) in patients with non–small-cell lung cancer^6,7,15,16,21,22,23,27^ and 19.4% (95% CI, 9.7%-29.1%) in patients with advanced gastric cancer.^10,13,19^ However, significant heterogeneity was noted (*I*^2^ ≥ 48.2%, *P* ≤ .001). There was only 1 study examining the incidence of HPD in squamous cell carcinoma of the head and neck (29.4%; 95% CI, 0.0%-6.5%),^9^ hepatocellular carcinoma (7.7%; 95% CI, 0.0%-6.5%),^31^ and melanoma (43.1%; 95% CI, 0.0%-6.5%).^24^ Ten studies included various types of cancer, which limited tumor type–based subgroup analysis.^5,8,11,12,14,17,18,25,26,28^

The answers for the 6 questions on the applicability, appropriateness, and clinical relevance of HPD definitions are described herein, and detailed evidence from the included studies is summarized in Table 3.

**Table 3.** Qualitative Review of the Questions and Detailed Evidence From the Included Studies

Source	Methods	Results	Answer
**1. Can HPD definitions be applied to most patients during ICI treatment?**			
Champiat et al,^5^ 2017	The number of patients initially recruited and the number of patients not evaluable for HPD and the reason were provided.	Among 218 recruited patients, 27 (12.4%) were not evaluable owing to the absence of prebaseline CT.	HPD definitions based on tumor growth acceleration could not be applied in many patients during ICI treatment (up to 39.1% of initially recruited patients) owing to the lack of required imaging studies.
Saada-Bouzid et al,^9^ 2017		Among 64 recruited patients, 25 (39.1%) were not evaluable owing to the absence of prebaseline CT (13 patients) or posttreatment CT (12 patients).	
Ferrara et al,^6^ 2018^a^		Among 249 recruited patients, 41 (16.5%) were not evaluable because images before or during treatment were not available.	
Ji et al,^12^ 2019		Among 45 recruited patients, 12 (26.7%) were excluded owing to no previous CT scan available before baseline.	
Kanjanapan et al,^14^ 2019		Among 352 recruited patients, 132 (37.5%) were excluded owing to lack of pretreatment CT scan.	
Kim et al,^7^ 2019		Among 379 recruited patients, 41 (10.8%) were excluded owing to absence of prebaseline CT scan (23 patients) or posttreatment CT scan (17 patients).	
Matos et al,^18^ 2020		Among 287 recruited patients, 22 (7.7%) had no CT scan before or after treatment.	
Tunali et al,^22^ 2019		Among 237 recruited patients, 5 (2.1%) had no prebaseline CT scan.	
**2. Is there any risk of overestimation or underestimation of HPD based on tumor kinetics assessment?**			
Kim et al,^7^ 2019	The correlation between SLD of target lesions and absolute difference in TGK ratio and TGR ratio was evaluated.	Heteroscedasticity phenomenon between absolute differences in log_2_ (ratio of TGK+1) and log_2_ (ratio of TGR+1) was prominent in patients with smaller baseline sum of longest diameters of the target lesions (Spearman correlation coefficient, −0.32; *P* = .001).	Defining HPD simply based on the ratio of tumor growth speed may result in a misinterpretation, as small absolute changes in volume or diameter may lead to greater variation if the baseline tumor burden is small or prebaseline tumor growth is slow.
Matos et al,^18^ 2020	Pre-TGR and post-TGR compared between HPD group and non-HPD progressor group, with HPD definition based on TGR.	HPD group showed significantly lower pre-TGR than that in non-HPD progressor group (*P* < .001).	
**3. Can HPD definitions appropriately reflect the change in overall tumor burden?**			
Kim et al,^15^ 2019	HPD was assessed by (1) volumetric approach in which the number of lesions to be assessed was not limited and any lesion that could be delineated on CT imaging was included in the analysis, and (2) RECIST-based target lesion–only approach.	When the 2 methods were compared, 22 of 135 (16.3%) discordant cases of HPD were found, and 9 of 48 (18.8%) patients with HPD by volumetric approach had exclusive progression of nontarget lesions with stable target lesions.	If HPD is assessed with target lesions only, tumor burden change may not be accurately reflected, which may lead to an underestimation of HPD.
Lo Russo et al,^16^ 2019	The appearance of new lesions in defining HPD and provided the detailed tumor growth profile were considered.	Among 39 patients with HPD, 35 (89.7%) had new lesions, and 10 (25.6%) showed progression by new lesion with stable target lesions.	
**Q4. Is PD defined by tumor response evaluation criteria (ie, RECIST 1.1 or iRECIST) necessary to define HPD?**			
Kanjanapan et al,^14^ 2019	Discordant cases of HPD by TGR calculation and by RECIST 1.1-defined PD were evaluated.	There were 4 patients with TGR>2 but stable disease on RECIST 1.1.	HPD assessment should not be confined to PD defined by tumor response evaluation criteria, as risk exists that significant tumor growth acceleration that would affect patients’ outcome within the boundary of non-PD would be missed if HPD is confined to PD.
Ten Berge et al,^21^ 2019	Prognosis compared between patients showing tumor growth acceleration (ie, increased TGR after start of therapy) and those showing tumor growth deceleration (ie, decreased TGR after start of therapy).	Regardless of the RECIST response categories, patients with tumor growth acceleration showed a significantly shorter median OS than those with tumor growth deceleration (median OS: 6.0 vs 18.0 mo; *P* = .002).	
**5. Is a time frame (ie, between prebaseline and baseline CT scan before treatment and between baseline and first follow-up) needed to define HPD, and if so, what is the optimal time frame?**			
Kato et al,^8^ 2017	Onset of HPD and/or survival of patients with HPD patients was reported.	HPD onset: range, 0.3-1.9 mo (0.3, 1.1, 1.5, 1.5, 1.7, and 1.9 mo); survival outcome was not provided	Time frame for posttreatment assessment should be explicitly set for prompt capture of HPD at an early point (possibly at ≤2 mo); if the posttreatment assessment time frame conformed to RECIST or was undefined, HPD would be underestimated or its detection would be delayed.
Hwang et al,^11^ 2020		HPD onset range, 0.8-2.7 mo; OS: median, 3.5 mo (95% CI, 2.6-4.4 mo)	
Ji et al,^12^ 2019		HPD onset: range, 0.94-1.4 mo (0.9, 1.2, 1.2, 1.4, and 1.4 mo); all patients with HPD patients died; OS: range, 2.4-7.4 mo (2.1, 3.6, 3.8, 5.6, and 7.4 mo)	
Kamada et al,^13^ 2019		HPD onset: range, 0.5-2.3 mo (0.5, 0.7, 1.2, 2.0, and 2.3 mo); 3 of 4 patients with HPD died within 3 mo (range, 0.7-2.1 mo [0.7, 1.8, 2.1 mo]), and 1 patient was alive at the study period.	
Petrioli et al,^25^ 2020		HPD onset: range, 1.8-2.3 mo (1.8, 2.1, and 2.3 mo); all patients with HPD died within 3.5 mo (range, 2.8-3.4 mo [2.8, 3.0, 3.4 mo])	
**6. Is HPD associated with clinical outcome by discriminating patients with HPD and with natural PD?**			
Champiat et al,^5^ 2017	Detailed results comparing the prognosis between patients with HPD and those with natural PD were provided.	No significant difference in OS between patients with HPD and those with natural PD (OS: 4.6 vs 7.6 mo [*P* = .19])	The reported outcome was heterogeneous across studies, raising questions regarding the clinical significance of HPD definitions. Further refinement and standardization of an HPD definition is required for identifying “clinically relevant” HPD.
Ferrara et al,^6^ 2018		Significantly shorter OS in patients with HPD and those with natural PD (HR, 2.18 [*P* = .003])	
Aoki et al,^10^ 2019		No significant difference in OS and PFS between patients with HPD and those with natural PD (OS: HR, 2.1 [*P* = .17]; PFS: HR, 1.1 [*P* = .76])	
Hwang et al,^11^ 2020		Significantly shorter OS in patients with HPD and those with natural PD (OS: 3.5 vs 7.3 mo [*P* < .001])	
Kim et al,^7^ 2019		Significantly shorter OS and PFS in patients with HPD and those with natural PD (OS: 1.6 vs 6.7 mo [*P* < .001]; PFS: 0.6 vs 1.6 mo [*P* < .001])	
Matos et al,^18^ 2020		For definition 1 (absolute size increase with new lesion considered), significantly shorter OS in patients with HPD and those with natural PD (median, 5.23 vs 7.33 mo; HR, 1.73 [*P* = .04]); for definition 2 (TGR only), no significant difference in OS (median, 4.2 vs 6.27 mo; HR, 1.4 [*P* = .35])	
Arasanz et al,^23^ 2020		Significantly shorter PFS in patients with HPD and those with natural PD (*P* = .04)	

Abbreviations: CT, computed tomography; HPD, hyperprogressive disease; HR, hazard ratio; ICI, immune checkpoint inhibitor; OS, overall survival; PD, progressive disease; PFS, progression-free survival; SLD, sum of longest diameters; TGK, tumor growth kinetics; TGR, tumor growth rate.

^a^ Data provided from a single institution.

First, can HPD definitions be applied to most patients during ICI treatment? To calculate the TGR ratio or TGK ratio, at least 3 radiologic examinations (prebaseline, baseline, and posttreatment) are required. Eight studies that used the TGR and/or TGK ratio to define HPD showed that 2.1% to 39.1% of patients were excluded because they did not have the required imaging studies (mostly prebaseline imaging). Therefore, HPD definitions based on the tumor growth acceleration could not be applied in a substantial portion of patients during ICI treatment.

Second, is there any risk of overestimation or underestimation of HPD based on tumor kinetics assessment? Kim et al^7^ detected a heteroscedasticity phenomenon, ie, the difference between the TGR and TGK ratios was prominent in patients with a smaller baseline sum of the longest diameters of the target lesions. In addition, Matos et al^18^ reported a significantly lower TGR in patients with HPD compared with patients with natural PD in the time between prebaseline and baseline CT scan before treatment onset, with the HPD definition as TGR ratio greater than 2. Therefore, because small absolute changes in volume or diameter may lead to greater variation if the baseline tumor burden is small or prebaseline tumor growth is slow, defining HPD based on the ratio of tumor growth speed may result in a misinterpretation.

Third, can HPD definitions appropriately reflect the change in overall tumor burden? In most of the included studies, new lesions appearing after ICI treatment or nonmeasurable lesions were not considered. According to Kim et al,^15^ HPD was assessed in 2 ways: a TGR-based volumetric approach in which the number of lesions to be assessed was not limited (any lesion that could be delineated on CT imaging was included in the analysis) and a TGR-based, target lesion–only approach. There were 16.3% discordant HPD cases between the 2 approaches, and 18.8% of hyperprogressors by the volumetric approach had exclusive progression of nontarget lesions with stable target lesions. Lo Russo et al^16^ included the appearance of new lesions as a part of the HPD definition and reported that 35 of 39 patients with HPD (89.7%) had new lesions and, among these patients, 10 (25.6%) showed progression by new lesions with stable target lesions. Hence, using HPD definitions that consider only target lesions, tumor burden change may not be accurately reflected, which may lead to an underestimation of HPD.

Fourth, is PD defined by tumor response evaluation criteria necessary to define HPD? In 10 studies, PD assessed by tumor response criteria at the first evaluation was a prerequisite to define HPD. According to Kanjanapan et al,^14^ 4 patients who showed a TGR ratio greater than 2 were not classified as having HPD because they had RECIST-defined stable disease. According to Ten Berge et al,^21^ regardless of the response categories, patients with tumor growth acceleration (increased TGR after treatment) showed significantly shorter OS than those with tumor growth deceleration (decreased TGR after treatment). Discrepancies exist between PD and tumor growth acceleration, and risk exists that significant tumor growth acceleration that would affect patients’ outcome within the boundary of non-PD would be missed if HPD is defined within the boundary of RECIST-defined PD. Therefore, HPD assessment should not be confined to PD defined by tumor response evaluation criteria.

Fifth, is a time frame needed to define HPD, and if so, what is the optimal time frame? Five studies reported the date of HPD onset and/or survival data for each patient with HPD. The earliest onset of HPD was 0.3 months, and the latest onset was 2.7 months; most HPD occurred within 2 months (average, 1.6 months). Most patients with HPD died within 3.5 months. Therefore, the time frame for posttreatment assessment should be specified for prompt capture of HPD at an early point (≤2 months). The time between baseline CT scan and first follow-up CT scan for response evaluation was 12 weeks or less and undefined in 12 studies; however, doing so would underestimate or delay the detection of HPD.

Sixth, is HPD associated with the clinical outcome by discriminating between patients with HPD vs natural PD? Defining HPD separately from PD would be more clinically meaningful if the outcome differs between patients with HPD vs natural PD than just if the outcome differs between patients with HPD and those without HPD. Seven studies compared the outcome between patients with HPD and those with natural PD, but the reported outcome was heterogeneous across studies, raising the question regarding the clinical significance of HPD definitions. Further refinement and standardization of an HPD definition is required for identifying clinically relevant HPD.

## Discussion

Hyperprogressive disease is currently regarded as a distinct outcome following ICI treatment.^1,2,4^ In our meta-analysis, the pooled incidence of HPD was 13.4%. However, the incidence and definition of HPD in each study were heterogeneous. The definitions of HPD could be divided into 4 categories, but each definition varied even within the same category. The pooled incidence of HPD also was altered according to the definition categories. This variation leads to concerns regarding the comparability of data across studies, difficulty with pooling, and poor clarity regarding which definition reflects the true aggressive tumor behavior.

Standardization and validation of an HPD definition is an important issue in immuno-oncology. Most studies adopted the idea of tumor growth acceleration based on either 3-dimensional tumor volume (TGR ratio) or 2-dimensional tumor diameter (TGK ratio) in which at least 3 specified times of imaging studies are prerequisites. Calculation of TGR ratio and TGK ratio also requires intensive measurements and many time interval calculations between CT scans to assess tumor growth kinetics. In a research setting, these approaches may be useful in demonstrating the paradoxical acceleration of tumor growth with ICI treatment. However, such complexity may be a challenge in incorporating the HPD concept into clinical practice, highlighting the need for a simpler and more readily available method to assess HPD.

Regarding the pooled incidence of HPD per its definition, it is rather straightforward that category 3 (early tumor burden increase) showed the highest pooled HPD incidence because it was the least strict criterion and it seems that the tumor burden increase criterion alone may be insufficient to define HPD in a clinically relevant way. Among the 5 studies included in category 3, 3 studies defined HPD as the tumor burden increase to RECIST-defined PD of 50% or 2-fold. These studies did not explicitly define the assessment interval after treatment initiation, but the time between baseline CT scan and first follow-up CT scan was at least 8 weeks. Among those studies, Forschner et al^24^ reported that disease-specific survival was significantly shorter in patients with vs without HPD, but none of the studies showed or they did not report whether there was a difference between patients with HPD and patients with natural PD.

Challenges have been presented regarding the current HPD definitions, and we categorized them into 6 questions. To summarize, HPD could be misjudged if the assessment was limited to the TGR or TGK ratio, target lesions, and RECIST-defined progressors, or if the assessment time frame conformed to RECIST. Results of clinical outcomes were heterogeneous on discriminating patients with HPD and those with natural PD, posing questions regarding the clinical meaningfulness of HPD definitions. As stated in our answer to question 1, diagnosing HPD based on TGR ratio and/or TKR ratio may preclude its clinical use in a substantial number of patients owing to the lack of prebaseline CT imaging data. This lack of data would become more problematic in patients with non–small-cell lung cancer and renal cell carcinoma for which ICIs have been approved as first-line therapy^42,43,44^ so that most ICI therapy is given to treatment-naive patients. Questions 2 and 3 are important, because inappropriate reflection of tumor growth acceleration and tumor burden may lead to misjudgment of HPD, and the prevailing concept of TGR ratio and/or TGK ratio of only the target lesions seems to have such risks. In particular, there were cases showing no change in tumor burden between the prebaseline and baseline CT scan period, followed by only slight increases in the period between the baseline and first follow-up CT scan, but the TGR ratio exceeded 2.^18^ Also, some cases showed stable target lesions while developing many new lesions that were therefore not captured if HPD is focused on only the target lesions.^15,16^ These issues should be considered when developing and validating a standardized HPD definition. As in question 6, distinguishing HPD from natural PD would be meaningful because there is growing evidence indicating that the outcome differs between patients with HPD and those with natural PD.^5,6,7,10,11,18,23^ Matos et al^18^ compared 2 definitions (absolute size increase with new lesion considered vs TGR only), and the former showed better discrimination between patients with HPD and those with natural PD by demonstrating a significant difference in OS compared with HPD definition based on TGR only. Also, because patients with HPD usually show rapid clinical deterioration early after ICI treatment, typically within 1 to 2 months, these patients may not undergo follow-up evaluation at 8 to 12 weeks. Thus, implementation of earlier disease assessment strategies and the integration of clinical deterioration would be crucial to identify patients with HPD.

The incidence of HPD according to tumor types was not significantly different with overlapping CIs for the 2 tumor types (15.0% in non–small-cell lung cancer and 19.4% in advanced gastric cancer), but adjustment according to the types of immunotherapeutic agents or other characteristics was not performed owing to the small number of studies and insufficient data. Further studies regarding the association between the type of tumors and HPD while considering covariates are anticipated.

We suggest several key requirements for an optimal definition of HPD. First, measurement of tumor growth acceleration based on tumor kinetics alone is insufficient to characterize HPD and, if maintained as a key factor, flexibility should be incorporated by combining other variables, such as clinical deterioration and a clearly defined time frame to assess tumor response. Time to failure within 2 months only might be too arbitrary and not sufficiently quantitative. Quantitative criteria based on Eastern Cooperative Oncology Group status or Karnofsky performance score should be developed. In the Response Assessment in Neuro-Oncology criteria for glioblastoma, clinical deterioration is incorporated and the Karnofsky performance score is used as a quantitative clinical measure for response evaluation. Second, a standardized measure of tumor growth acceleration including assessment of target lesions, nontarget or nonmeasurable lesions, and new lesions should be established. The measurement should also be easy to perform and readily available without sophisticated software; calculation based on tumor diameter might be advantageous to tumor volume. Also, the cutoff values for HPD diagnosis should be meticulously determined based on large-scale data. Third, alternative diagnostic criteria are required for patients without pretreatment imaging studies; these criteria should be based on images acquired at baseline and first follow-up and show equivalent diagnostic performance with the definitions based on tumor growth acceleration derived from 3 time points.

### Limitations

Our study has limitations. First, the number of included studies is relatively small, and most were retrospective analyses. Further large-scale prospective studies are necessary. Second, publication bias was present in pooling the incidence of HPD, probably owing to small-study effects. Also, the difference in HPD definitions across studies might have led to heterogeneity in the pooled HPD incidence. Because most studies were retrospective, the TGR or TGK assessment cannot be validated in published clinical trials since the prebaseline CT imaging data were not captured.

## Conclusions

We divided the diverse definitions of HPD across the included studies into 4 categories. The pooled incidence of HPD was 13.4% but varied from 5.9% to 43.1%. Hyperprogressive disease could be overestimated or underestimated based on current assessment. Varying incidence and the definition of HPD and challenges of current assessment of HPD indicate the need for establishing uniform and clinically relevant criteria based on currently available evidence.

## Notes

Supplement. **eTable 1.** Detailed Search Strategy **eTable 2.** Characteristics of the Conference Abstracts (n=29) **eTable 3.** The Newcastle-Ottawa Scale (NOS) Quality Assessment of the Enrolled Studies **eTable 4.** Subgroup Analyses Regarding Definition of HPD and Type of Tumor **eFigure 1.** Flow Diagram of the Conference Abstract Selection Process **eFigure 2.** Funnel Plot Indicating Substantial Publication Bias (*P*=.003) **eFigure 3.** Pooled Incidence of HPD in Studies With Newcastle-Ottawa Scale Score ≥7
