# Supplementary material for: Definition, Incidence, and Challenges for Assessment of Hyperprogressive Disease During Cancer Treatment With Immune Checkpoint Inhibitors: A Systematic Review and Meta-analysis
Source: JAMA Netw Open. 2021 Mar 24;4(3):e211136. doi: 10.1001/jamanetworkopen.2021.1136 (PMC7991969; doi:10.1001/jamanetworkopen.2021.1136)
Supplement: Supplement. — eTable 1. Detailed Search Strategy eTable 2. Characteristics of the Conference Abstracts (n=29) eTable 3. The Newcastle-Ottawa Scale (NOS) Quality Assessment of the Enrolled Studies eTable 4. Subgroup Analyses Regarding Definition of HPD and Type of Tumor eFigure 1. Flow Diagram of the Conference Abstract Selection Process eFigure 2. Funnel Plot Indicating Substantial Publication Bias (P=.003) eFigure 3. Pooled Incidence of HPD in Studies With Newcastle-Ottawa Scale Score ≥7 [file jamanetwopen-e211136-s001.pdf]

Supplementary Online Content

Park HJ, Kim KW, Won SE, et al. Definition, incidence, and challenges for assessment of hyperprogressive disease during cancer treatment with immune checkpoint inhibitors: a systematic review and meta-analysis. *JAMA Netw Open*. 2021;4(3):e211136. doi:10.1001/jamanetworkopen.2021.1136

eTable 1. Detailed Search Strategy

eTable 2. Characteristics of the Conference Abstracts (n=29)

eTable 3. The Newcastle-Ottawa Scale (NOS) Quality Assessment of the Enrolled Studies

eTable 4. Subgroup Analyses Regarding Definition of HPD and Type of Tumor

eFigure 1. Flow Diagram of the Conference Abstract Selection Process

eFigure 2. Funnel Plot Indicating Substantial Publication Bias (P =.003)

eFigure 3. Pooled Incidence of HPD in Studies With Newcastle-Ottawa Scale Score ≥7

This supplementary material has been provided by the authors to give readers additional information about their work.

© 2021 Park HJ et al. *JAMA Network Open.*

eTable 1. Detailed search strategy

Database	Search strategy
MEDLINE	(checkpoint OR 'check point' OR check OR 'immunotherapy'/exp OR immunotherapy OR 'pd 1'/exp OR 'pd 1' OR 'pd l1' OR 'ctla 4'/exp OR 'ctla 4' OR 'ipilimumab'/exp OR ipilimumab OR 'nivolumab'/exp OR nivolumab OR 'pembrolizumab'/exp OR pembrolizumab OR 'atezolizumab'/exp OR atezolizumab OR 'avelumab'/exp OR avelumab OR 'durvalumab'/exp OR durvalumab) AND hyperprogress* AND [1-1-0001]/sd NOT [3-3-2020]/sd
EMBASE	("checkpoint"[Text Word] OR "check-point"[Text Word] OR "check"[Text Word] OR "immunotherapy"[Text Word] OR ("PD-1"[Text Word] OR "PD-L1"[Text Word] OR "CTLA-4"[Text Word] OR "ipilimumab"[Text Word] OR "nivolumab"[Text Word] OR "pembrolizumab"[Text Word] OR "atezolizumab"[Text Word] OR "avelumab"[Text Word] OR "durvalumab"[Text Word])) AND "hyperprogress*"[Text Word] AND 0001/01/01:2020/03/03[Date - Publication]

© 2021 Park HJ et al. *JAMA Network Open.*

eTable 2. Characteristics of the conference abstracts (n=29)

Study	Study design	Tumor	Agent(s)	No. of previous treatment lines	HPD definition	No. of patients	Incidence of HPD	Pre-treatment period	Post-treatment period	Prognostic impact of HPD	
										HPD vs non-HPD	HPD vs PD without HPD
Alfieri S et al (2019)	Retrospective study	SqCC of head and neck	ICI [a]	≥2 (75%)	PD by RECIST 1.1 at first evaluation and TGK_{post}/TGK_{pre} ≥2	88	8.0% (7/88)	NA	NA	OS, 3.7 vs 8.3 months (P = 0.348), PFS, 1.8 vs 3.5 months (P = 0.001)	NA
Ayala De Miguel P et al (2019)	Retrospective study	NSCLC	ICI monotherapy [a]	0 (18%), ≥1 (82%)	PD by RECIST 1.1 at first evaluation and ΔTGR > 50% per month	66	10.6% (7/66)	NA	< 2 months	OS, HR, 4.35; (43.6 vs 11.3 months) (P = 0.0037)	NA
Colle E et al (2019)	Retrospective study	Melanoma	PD-1 inhibitor monotherapy	0 (47%)	progression/death within 3 months with normal initial LDH and ECOG at baseline, and either ECOG increased from 0 to 3-4, either LDH increased from normal to elevated or both	793	10.3% (82/793)	NA	NA	NA	NA
Economopoulou P et al (2019)	Retrospective study	SqCC of head and neck	PD-1 or PD-L1 inhibitor monotherapy	NA	Radiological HPD (TGKR≥2) or Clinical HPD (Disease-related rapid clinical deterioration post IO)	62	25.8% (16/62)	NA	3 months	NA	NA
Farè E et al (2018)	Retrospective study	Miscellaneous advanced solid tumors	PD-1 inhibitor monotherapy or combined with PD-L1 inhibitor	NA	PD by RECIST 1.1 at first evaluation and TGR_{POST}/TGR_{PRE} ≥ 2	197	3.6% (7/197)	2 weeks to 3 months	NA	NA	NA
Feng Y et al (2018)	Retrospective analysis of clinical trial data	AGC	Nivolumab	≥ 2	Definition 1: An increase of ≥20% in the sum of longest diameter (SLD) of target lesions at 8 weeks post baseline	243	27.6%	NA	8 weeks	NA	NA
					Definition 2: An increase of ≥50% in the sum of longest diameter (SLD) of target lesions at 8 weeks post baseline	243	5.4%	NA	8 weeks	NA	NA
					Definition 3: An increase of	243	1.2%	NA	8 weeks	NA	NA

© 2021 Park HJ et al. *JAMA Network Open.*

					≥100% in the sum of longest diameter (SLD) of target lesions at 8 weeks post baseline						
Freixinos VR et al (2018)	Retrospective analysis of clinical trial data	Gynecologic cancers[b]	ICI [a]	0-8	≥40% tumor burden increase or ≥20% plus multiple new lesions	60	23%	NA	NA	NA	NA
Gandara DR et al (2018)	Retrospective analysis of clinical trial data	NSCLC	Atezolizumab	≥ 1	≥ 50% increase in the SLD (per investigator) from baseline to first assessment (6 weeks) or death due to PD per investigator within 12 weeks without a post-tx scan	425	10.4% (44/425)	NA	NA	NA	NA
Ghiglione L et al (2019)	Retrospective study	Various [c]	ICI (mono or dual therapy) or combined with chemotherapy [a]	≥ 2 (24%)	$TGR_{POST}/TGR_{PRE} \geq 2$	187	9.6% (18/187)	NA	NA	OS, HR, 2.11; 4.65 vs. 11.3 months (*P* < 0.005) PFS, 2.25 vs 3.97 months (*P* < 0.001)	NA
Giusti R et al (2019)	Retrospective study	NSCLC	PD-1 inhibitor monotherapy	0	TTF < 2 months, > 50% increase in tumor burden compared with pre-immunotherapy imaging	20	25% (5/20)	NA	NA	NA	NA
Gomez LG et al (2019)	Retrospective study	NSCLC	PD-1 inhibitor monotherapy	NA	$TGK_{post}/TGK_{pre} \geq 2$	42	14%	NA	NA	NA	NA
Han J et al (2019)	Retrospective study	NSCLC	ICI [a]	NA	ΔTGR > 50%	51	11.8% (6/51)	NA	NA	NA	NA
Honjo O et al (2018)	Retrospective study	Lung cancer	PD-1 inhibitor monotherapy	NA	PD by RECIST 1.1 at first evaluation and $TGR_{POST}/TGR_{PRE} \geq 2$	216	3.2% (7/216)	NA	NA	OS, 175 days (IQR, 54–618) and 141 days (IQR, 22–635)	NA
Kanjanapan Y et al (2018)	Retrospective analysis of clinical trial data	Various d	ICI monotherapy or combined with co-stimulatory molecules [a]	0-7	PD by RECIST 1.1 at first evaluation and $TGR_{POST}/TGR_{PRE} \geq 2$	182	7.1% (13/182)	NA	NA	OS, HR, 0.75 (non-HPD as reference, *P* = 0.5)	NA

© 2021 Park HJ et al. *JAMA Network Open.*

Kim J et al (2019)	Retrospective study	NSCLC	ICI [a]	NA	$TGK_{post}/TGK_{pre} \geq 2$ and TTF < 2 months	231	10.8% (25/231)	NA	NA	OS, 5.6 vs 7.4 months (*P* < 0.001)	NA
Lee JC et al (2019)	Retrospective study	NSCLC	PD-1 inhibitor monotherapy	1 (100%)	PD by RECIST 1.1 at first evaluation and $TGR_{POST}/TGR_{PRE} \geq 2$	83	19.3% (16/83)	NA	NA	NA	OS, 2.2 months (95% CI, 0.92 to 3.75) vs 4.1 months (95% CI, 1.54 to 6.67)
Lo Russo G et al (2019)	Retrospective study	NSCLC	ICI [a]	NA	Fulfilling 3 or more of the followings: (1) TTF < 2 months, (2) ≥ 50% increase of tumor burden between baseline and first evaluation, (3) ≥ 2 new lesions in an organ already involved between baseline and first evaluation, (4) disease spread to a new organ between baseline and first evaluation, and (5) decrease in ECOG performance status ≥ 2 during the first 2 months of treatment	257	20.6% (53/257)	NA	8 weeks	NA	OS, HR, 2.481 (*P* < 0.0001), PFS, HR, 2.448 (*P* < 0.0001)
Park C et al (2019)	Retrospective study	NSCLC	PD-1 inhibitor or PD-L1 inhibitor monotherapy	1 (100%)	Definition 1: $TGR_{POST}/TGR_{PRE} >2$	73	12.3% (9/73)	NA	NA	NA	OS, 2.4 vs 5.2 months (*P* = 0.002), PFS, 1.6 vs 2.1 months (*P* < 0.001)
					Definition 2: $TGK_{post}/TGK_{pre} \geq 2$	73	15.1% (11/73)		NA	NA	OS, 2.4 vs 5.2 months (*P* = 0.002), PFS, 1.6 vs 2.1 months (*P* < 0.001)
					Definition 3: $\Delta TGR > 50\%$	73	0% (0/73)		NA	NA	NA
Patil P et al (2018)	Retrospective study	NSCLC	PD-1 inhibitor or PD-L1 inhibitor monotherapy	NA	$TGK_{POST}/TGK_{PRE} \geq 2$	336	8.3% (28/336)	NA	NA	NA	NA
Perna M et al	Retrospective study	NSCLC	PD-1 inhibitor monotherapy	≥ 1	> 50% increase in tumor burden compared with pre-	46	2.2% (1/46)	NA	NA	NA	NA

© 2021 Park HJ et al. *JAMA Network Open.*

(2018)					immunotherapy imaging						
Simões Da Rocha PF et al (2018)	Retrospective study	NSCLC	PD-1 inhibitor monotherapy	NA	$TGR_{POST}/TGR_{PRE} \geq 2$	40	30% (12/40)	NA	NA	NA	NA
Suarez C et al (2019)	Retrospective analysis of clinical trial data	Urothelial carcinoma, RCC	ICI monotherapy or combination [a]	≥ 1 (53%)	PD by RECIST at first 8 weeks after treatment initiation and minimum increase in the measurable lesions of 10 mm plus: (1) 40% increase in STL compared with baseline and/or (2) 20% increase in STL compared with baseline plus the appearance of new lesions in at least two different organs	88	10.2% (9/88)	NA	NA	OS, 8.87 vs 4.77 months (*P* = 0.065)	NA
Sugimoto N et al (2018)	Retrospective study	AGC	PD-1 inhibitor monotherapy	NA	$TGK_{POST}/TGK_{PRE} \geq 2$	9	55.6% (5/9)	NA	NA	NA	NA
Sunakawa Y et al (2019)	clinical trial (inter-rim analysis)	AGC	PD-1 inhibitor monotherapy	NA	$TGR_{POST}/TGR_{PRE} \geq 2$	105	24.8% (26/105)	NA	NA	NA	NA
Suzuki T et al (2020)	Retrospective study	AGC	PD-1 inhibitor monotherapy	NA	$TGR_{POST}/TGR_{PRE} \geq 2$	218	17.4% (38/218)	NA	NA	NA	OS, HR, 1.0; 5.0 vs 4.6 months (p = 0.8695), PFS, HR, 1.3; 1.5 vs 1.6 months (*P* = 0.1194)
Takahashi R et al (2019)	Retrospective study	NSCLC	PD-1 or PD-L1 inhibitor monotherapy	NA	TTF < 1 month and $TGK_{post}/TGK_{pre} \geq 2$	94	4.3% (4/94)	NA	1 month	NA	NA
Tan TJY et al (2019)	Retrospective analysis of clinical trial data	Triple-negative breast cancer	IO monotherapy/combination or combined with chemotherapy [a]	0-8	$TGR_{POST}/TGR_{PRE} \geq 2$	40	10% (4/40)	NA	NA	OS, HR, 0.89 (non-HPD as reference, *P* = 0.41)	NA
Tang B et al (2019)	Retrospective analysis of	Melanoma	PD-1 inhibitor monotherapy	NA	PD by RECIST 1.1 at first evaluation and	90	5.6% (5/90)	NA	NA	NA	NA

© 2021 Park HJ et al. *JAMA Network Open.*

	clinical trial data				$TGR_{POST}/TGR_{PRE} \geq 2$						
Zalcman G et al (2019)	clinical trial	Malignant pleural mesothelioma	PD-1 inhibitor mono or combined with PD-L1 inhibitor	1-2	PD by RECIST 1.1 at first evaluation and $TGK_{POST}/TGK_{PRE} \geq 2$	187	5.9% (11/187)	NA	NA	NA	OS, HR, 0.37 (*P* = 0.006)

Abbreviations: AGC, advanced gastric cancer; ECOG, Eastern Cooperative Oncology Group; HPD, hyperprogressive disease; HR, hazard ratio; IO, immunotherapy; IQR, interquartile range; NA, not avaiable; NSCLC, non-small cell lung cancer; OS, overall survival; PD, progressive disease; PFS, progression-free survival; POST, post-treatment assessment period; PRE, pre-treatment assessment period; RCC, renal cell carcinoma; SqCC, squamous cell carcinoma; STL, sum of target lesions; TGK, tumor growth kinetics; TGR, tumor growth ratio

[a] Details of the used agent(s) were not provided.

[b] Ovarian cancer (n=32), endometrial cancer (n=8), cervical cancer (n=15) and vulvar cancer (n=5)

[c] NSCLC (62%), urothelial carcinoma (23%) and kidney carcinoma (15%)

[d] Head and neck (18%), gynecological (16%), lung (15%), gastrointestinal (15%), genitourinary (12%), melanoma (8%), sarcoma (7%), endocrine (5%) and breast (4%) cancers

© 2021 Park HJ et al. *JAMA Network Open.*

eTable 3. The Newcastle-Ottawa scale (NOS) quality assessment of the enrolled studies

Study	Selection of cohorts				Comparability of cohorts	Outcome		
	Representativeness of the exposed cohort [a]	Selection of the non-exposed cohort	Ascertainment of exposure	Demonstration that outcome of interest was not present at start of study [b]	Comparability of cohorts on the basis of the design or analysis [c]	Ascertainment of outcome	Adequate follow-up [d]	Adequacy of follow-up of cohorts [e]
Champiat S *et al* (2017)	☆	☆	☆		☆☆	☆	☆	☆
Kato S *et al* (2017)	☆	☆	☆		☆☆	☆		
Saada-Bouzid E *et al* (2017)	☆	☆	☆		☆☆	☆	☆	☆
Ferrara R *et al* (2018)	☆	☆	☆		☆☆	☆	☆	☆
Abbas W *et al* (2019)	☆	☆	☆			☆		
Aoki M *et al* (2019)	☆	☆	☆		☆☆	☆	☆	☆
Hwang I *et al* (2019)	☆	☆	☆		☆☆	☆	☆	☆
Ji Z *et al* (2019)	☆	☆	☆		☆☆	☆		
Kamada T *et al* (2019)	☆	☆	☆			☆		
Kanjanapan Y *et al* (2019)	☆	☆	☆		☆☆	☆	☆	☆
Kim CG *et al* (2019)	☆	☆	☆		☆☆	☆	☆	☆
Kim Y *et al* (2019)	☆	☆	☆		☆☆	☆	☆	☆
Lo Russo G *et al* (2019)	☆	☆	☆		☆☆	☆	☆	☆
Lu Z *et al* (2019)	☆	☆	☆	☆	☆☆	☆	☆	☆
Matos I *et al* (2019)	☆	☆	☆		☆☆	☆	☆	☆
Sasaki A *et al* (2019)	☆	☆	☆		☆☆	☆	☆	☆
Scheiner B *et al* (2019)	☆	☆	☆			☆	☆	☆
Ten Berge D *et al* (2019)	☆	☆	☆			☆	☆	☆
Tunali I *et al* (2019)	☆	☆	☆			☆	☆	☆

© 2021 Park HJ et al. *JAMA Network Open.*

Arasanz H *et al* (2020)	☆	☆	☆	☆	☆	☆	☆	☆
Forschner A *et al* (2020)	☆	☆	☆		☆☆	☆	☆	☆
Petrioli R *et al* (2020)	☆	☆	☆			☆		
Refae S *et al* (2020)	☆	☆	☆		☆☆	☆	☆	☆
Ruiz-Patino A *et al* (2020)	☆	☆	☆		☆	☆		

Each study could be awarded a maximum of nine stars: a maximum of two stars for the item regarding comparability and a maximum of one star for other 7 items.

[a] Exposure was the occurrence of hyperprogressive disease. All included studies were awarded one star because patients with hyperprogression were truly or somewhat representative and not selected from general population with hyperprogression.

[b] One star was awarded if a study was a prospective cohort study.

[c] A maximum of two stars could be awarded for this item. If a study adjusted for baseline demographic factors (e.g., age, gender), one star was awarded, and if a study adjusted for additional confounding factors (e.g., ECOG status, Royal Marsden Hospital score, histology, number of metastasis site, drug type, other laboratory data, etc.), an additional star was awarded.

[d] For studies reporting OS or PFS, with comparison between hyperprogressors and non-hyperprogressors or between hyperprogressors and progressors without hyperprogression, one star was awarded.

[e] If a study reported a follow up rate of ≥ 80%, one score was awarded.

© 2021 Park HJ et al. *JAMA Network Open.*

eTable 4. Subgroup analyses regarding definition of HPD and type of tumor.

Factor	Pooled incidence of HPD (%)	P value			
**HPD definition**		vs Category 1	vs Category 2	vs Category 3	vs Category 4
Category 1 (TGR ratio)	9.4 (6.9–12.0)	-	0.556	0.064	0.883
Category 2 (TGK ratio)	15.8 (8.0–23.7)	0.556	-	0.918	0.896
Category 3 (early tumor burden increase)	20.6 (9.3–31.8)	0.064	0.918	-	0.370
Category 4 (combination)	12.1 (7.3–17.5)	0.883	0.896	0.370	-
**Type of tumor**		0.441			
NSCLC	15.0 (10.5–19.5)				
AGC	19.4 (9.7–29.1)				

Abbreviation: AGC, advanced gastric cancer; HPD, hyperprogressive disease; NSCLC, non-small cell lung cancer, TGK, tumor growth kinetics; TGR, tumor growth ratio.

© 2021 Park HJ et al. *JAMA Network Open.*

eFigure 1. Flow diagram of the conference abstract selection process

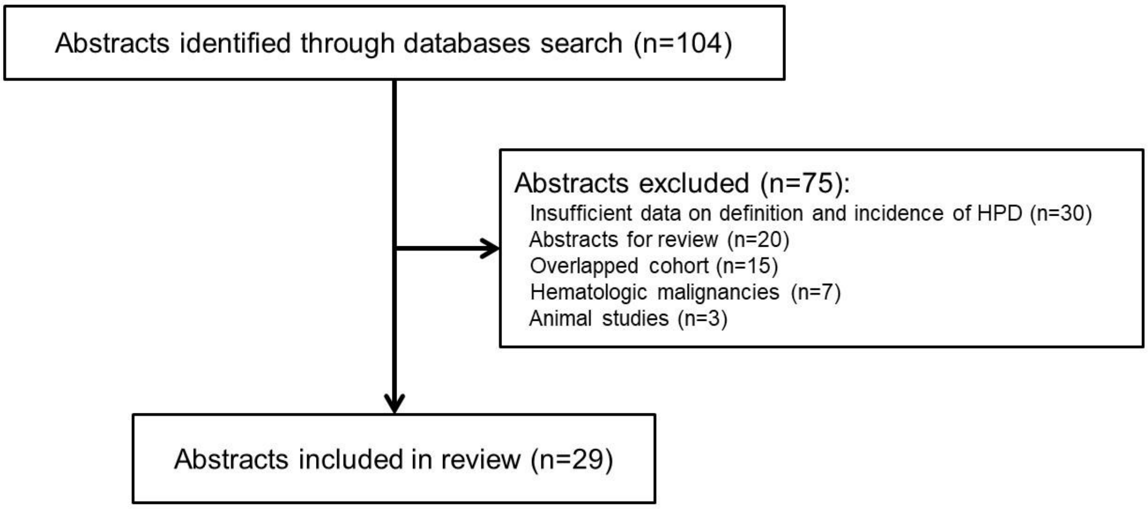

© 2021 Park HJ et al. *JAMA Network Open.*

eFigure 2. Funnel plot indicating substantial publication bias (*P* =.003)

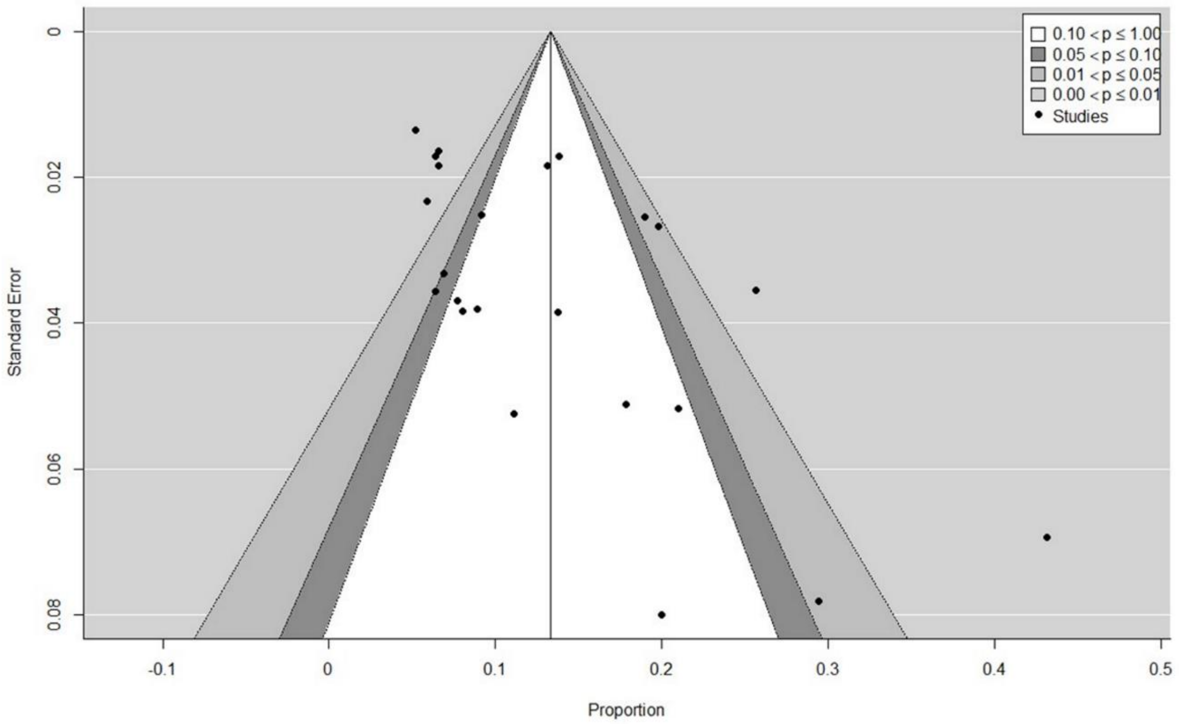

© 2021 Park HJ et al. *JAMA Network Open.*

eFigure 3. Pooled incidence of HPD in studies with Newcastle-Ottawa Scale score ≥7

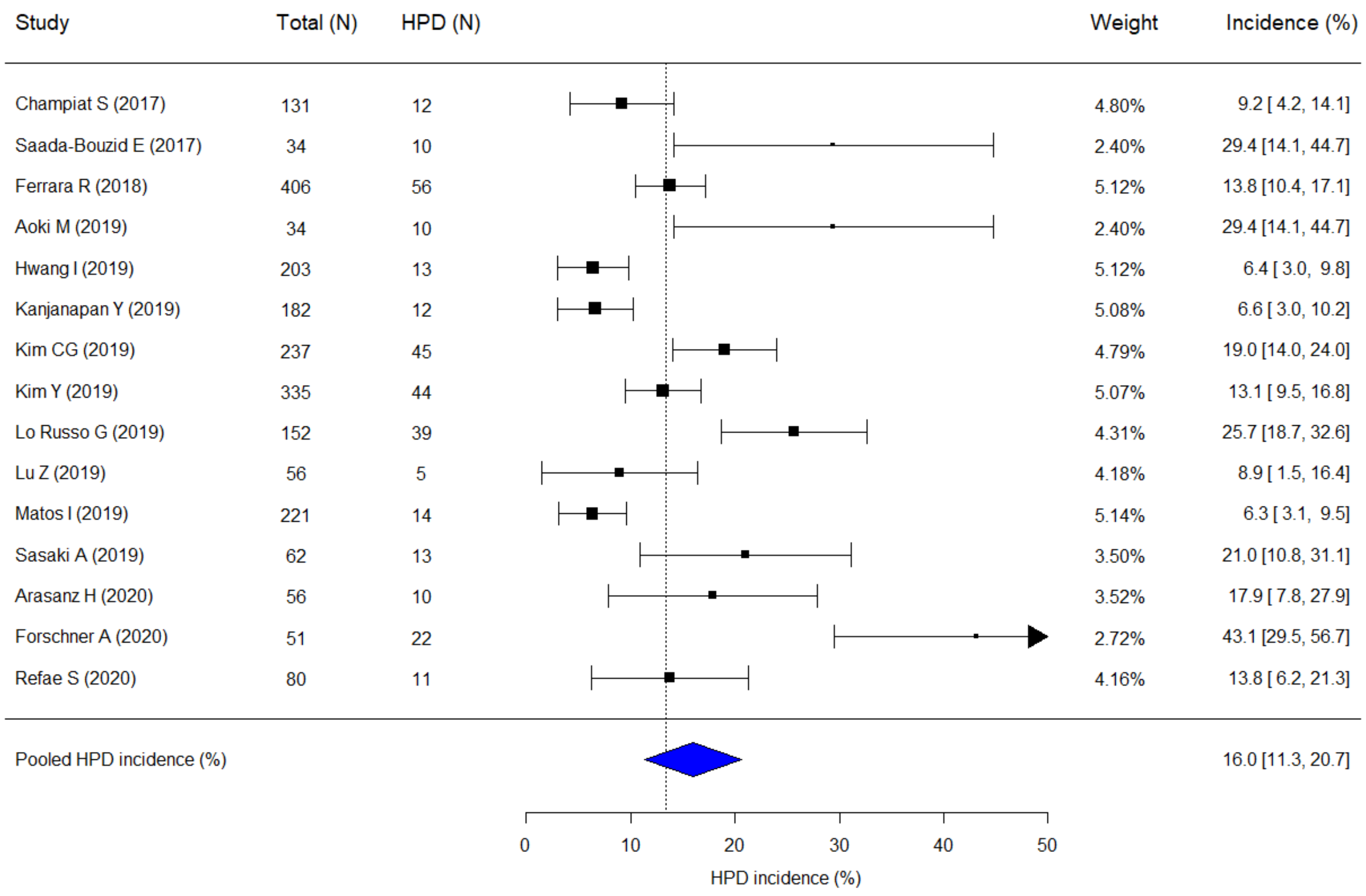

Fifteen studies out of 24 included studies (62.5%) were awarded equal or higher than 7 scores using the Newcastle-Ottawa Scale. The pooled incidence of HPD from the 15 studies was 16.0% (95% CI, 11.3–20.7%).

© 2021 Park HJ et al. *JAMA Network Open.*
